# Supplementary material for: Deep Learning Algorithms for Diagnosis of Lung Cancer: A Systematic Review and Meta-Analysis
Source: Cancers (Basel). 2022 Aug 9;14(16):3856. doi: 10.3390/cancers14163856 (PMC9405626; doi:10.3390/cancers14163856)
Supplement: Supplementary file 1 [file cancers-14-03856-s001.zip › Supplementary File S1.pdf]

Supplementary File S1. Database Search Strategies

("artificial intelligence" OR "deep learning" OR "machine learning" OR "computer-aided" OR CAD OR "neural network*" OR CNN OR "convolutional neural network*") AND (“lung cancer” OR “lung nodule” OR “lung cancer screening”) AND (CT OR LDCT OR “computed tomography”)
